# Supplementary material for: Knowledge syntheses in medical education: Meta-research examining author gender, geographic location, and institutional affiliation
Source: PLoS One. 2021 Oct 26;16(10):e0258925. doi: 10.1371/journal.pone.0258925 (PMC8547645; doi:10.1371/journal.pone.0258925)
Supplement: S1 Appendix — (DOCX) [file pone.0258925.s001.docx]

Supplemental Appendix 1: List of Core Journals and Search Strategy

Core medical education journals (n=14) included:

*Academic Medicine, Advances in Health Sciences Education, BMC Medical Education, Canadian Medical Education Journal, Clinical Teacher, International Journal of Medical Education, Advances in Medical Education and Practice, Journal of Graduate Medical Education, Medical Education, Medical Education Online, Medical Teacher, Perspectives on Medical Education, Teaching and Learning in Medicine*, and *The Journal of Continuing Education in the Health Professions*.

Search Strategy

**PubMed Search String (LIMIT: 1999/01/01 – 2019/12/31)**

("knowledge synthesis" [title/abstract] OR "literature review"[title/abstract] OR "evidence synthesis" [title/abstract] OR "systematic review"[title/abstract] OR review[title] OR "meta-analysis" [Publication Type] OR review [Publication Type] OR "systematic"[sb] OR scoping[title/abstract] OR "meta-synthesis"[title/abstract] OR "narrative review" [title/abstract] OR "critical review"[title/abstract] OR "critical synthesis"[title/abstract] OR "integrative review" [title/abstract] OR "integrative synthesis"[title/abstract] OR "qualitative review"[title/abstract] OR "metastudy"[title/abstract] OR "realist review"[title/abstract] OR "rapid review"[title/abstract] OR "umbrella review" [title/abstract] OR "BEME" [title/abstract] OR “consensus conference”[title/abstract] OR "medline" [title/abstract] OR "cinahl"[title/abstract] OR "PubMed"[title/abstract] OR "embase"[title/abstract] OR "psycInfo"[title/abstract]) AND ("Acad Med"[journal] OR "Adv Health Sci Educ Theory Pract"[journal] OR "Adv Med Educ Pract"[journal] OR "BMC Med Educ"[journal] OR "Can Med Educ J"[journal] OR “Clin Teach"[journal] OR "J Contin Educ Health Prof"[journal] OR "Teach Learn Med"[journal] OR "Perspect Med Educ"[journal] OR "Med Educ"[journal] OR "Med Educ Online"[journal] OR "Med Teach"[journal] OR "J Grad Med Educ"[journal] OR "Int J Med Educ"[journal])

**PubMed Search String for All Citations (LIMIT: 1999/01/01 – 2019/12/31)**

("Acad Med"[journal] OR "Adv Health Sci Educ Theory Pract"[journal] OR "Adv Med Educ Pract"[journal] OR "BMC Med Educ"[journal] OR "Can Med Educ J"[journal] OR “Clin Teach"[journal] OR "J Contin Educ Health Prof"[journal] OR "Teach Learn Med"[journal] OR "Perspect Med Educ"[journal] OR "Med Educ"[journal] OR "Med Educ Online"[journal] OR "Med Teach"[journal] OR "J Grad Med Educ"[journal] OR "Int J Med Educ"[journal])

**Web of Science (LIMIT: 1999/01/01 – 2019/12/31)**

((TI=("knowledge synthesis") OR AB=("knowledge synthesis")) OR (TI=("evidence synthesis") OR AB=("evidence synthesis")) OR (TI=("literature review") OR AB=("literature review")) OR

(TI=("evidence synthesis") OR AB=("evidence synthesis")) OR (TI=("systematic review") OR AB=("systematic review") OR (TI=”(review) OR AB=(review)) OR (DT=(review)) OR

(TI=(scoping) OR AB=(scoping)) OR (TI=("meta-synthesis") OR AB=("meta-synthesis")) OR

(TI=("narrative review") OR AB=("narrative review")) OR (TI=("critical review") OR AB=("critical review")) OR (TI=("critical synthesis") OR AB=(""critical synthesis")) OR (TI=("integrative review") OR AB=("integrative review")) OR (TI=("integrative synthesis") OR AB=("integrative synthesis")) OR (TI=("qualitative review") OR AB=("qualitative review")) OR (TI=("metastudy") OR AB=("metastudy")) OR (TI=("realist review") OR AB=("realist review")) OR (TI=("critical review") OR AB=("critical review")) OR (TI=("critical review") OR AB=("critical review")) OR (TI=("rapid review") OR AB=("rapid review")) OR (TI=("umbrella review") OR AB=("umbrella review")) OR (TI=("BEME") OR AB=("BEME")) OR (TI=(“consensus conference”) OR AB=(“consensus conference”)) OR (TI=("medline") OR AB=("medline")) OR (TI=("cinahl") OR AB=("cinahl")) OR (TI=("pubmed") OR AB=("pubmed")) OR (TI=("embase") OR AB=("embase")) OR (TI=("psychinfo") OR AB=("psychinfo"))) AND SO=((Advances in Health Sciences Education) OR (Clinical Teacher) OR (Teaching "and" Learning in Medicine) OR (Medical Education Online) OR (Medical Teacher))
