# Supplementary material for: Knowledge syntheses in medical education: Meta-research examining author gender, geographic location, and institutional affiliation
Source: PLoS One. 2021 Oct 26;16(10):e0258925. doi: 10.1371/journal.pone.0258925 (PMC8547645; doi:10.1371/journal.pone.0258925)
Supplement: S2 Appendix — (DOCX) [file pone.0258925.s002.docx]

Supplemental Appendix 2: Top 10 most prolific authors overall, first authors, and last authors in publishing knowledge syntheses (KS) in 14 core medical education journals between 1999-2019.

| Most prolific overall authors (*gender) | Number of Knowledge Synthesis | Most prolific first authors *(gender) | Number of Knowledge Synthesis | Most prolific last authors (*gender) | Number of Knowledge Synthesis |
| --- | --- | --- | --- | --- | --- |
| Cook, David (M) | 36 | Cook, David (M) | 21 | Cook, David (M) | 9 |
| ten cate, Olle (M) | 15 | Mcgaghie, William (M) | 6 | Oswald, Anna (F) | 6 |
| Hatala, Rose (F) | 12 | Gordon, Morris (M) | 5 | ten cate, Olle (M) | 6 |
| Scherpbier, Albert (M) | 12 | Norman, Geoff (M) | 5 | Dornan, Todd (M) | 4 |
| Mcgaghie, William (M) | 11 | Maudsley, Gillian (F) | 4 | Durning, Steven (M) | 4 |
| Brydges, Ryan. (M) | 11 | Hauer, Karen (F) | 4 | Hatala, Rose (F) | 4 |
| Van Der Vleuten, Cees (M) | 11 | Burgess, Annette (F) | 4 | Krishna, Lalit (M) | 4 |
| Dornan, Todd (M) | 11 | Brydges, Ryan (M) | 4 | Van Der Vleuten, Cees (M) | 4 |
| Eva, Kevin (M) | 10 | Buckley, Sharon (F) | 3 | Archer, Julian (M) | 3 |
| Thistlethwaite, Jill (F) | 9 | Benbassat, Jochanan (M) | 3 | Bordage, Georges (M) | 3 |
|  |  |  |  | Charlin, Bernard (M) | 3 |
|  |  |  |  | Fernandez, Rosemarie (F) | 3 |
|  |  |  |  | Horsley, Tanya (F) | 3 |
|  |  |  |  | Hu, Wendy (F) | 3 |
|  |  |  |  | Khan, Khalid (M) | 3 |
|  |  |  |  | Lingard, Lorelei (F) | 3 |
|  |  |  |  | Maloney, Stephen (M) | 3 |
|  |  |  |  | McKelvy, Dina (F) | 3 |
|  |  |  |  | Mellis, Craig (M) | 3 |
|  |  |  |  | O'Sullivan, Patricia (F) | 3 |
|  |  |  |  | Reeves, Scott (M) | 3 |
|  |  |  |  | Roberts, Trudie (F) | 3 |
|  |  |  |  | Scalese, Ross (M) | 3 |
|  |  |  |  | Scherpbier, Albert (M) | 3 |
|  |  |  |  | Schuwirth, Lambert (M) | 3 |
|  |  |  |  | Thistlethwaite, Jill (F) | 3 |
|  |  |  |  | van Merrienboer, Jeroen (M) | 3 |
|  |  |  |  | Violato, Claudio (M) | 3 |
|  |  |  |  | Wayne, Diane (F) | 3 |
|  |  |  |  | Wieringa-de Waard, Margreet (F) | 3 |
|  |  |  |  | Williams,Brett (M) | 3 |

*Gender: M=male, F=female
