# Supplementary material for: Knowledge syntheses in medical education: Meta-research examining author gender, geographic location, and institutional affiliation
Source: PLoS One. 2021 Oct 26;16(10):e0258925. doi: 10.1371/journal.pone.0258925 (PMC8547645; doi:10.1371/journal.pone.0258925)
Supplement: Attachment — Submitted filename: Response to Reviewers.docx [file pone.0258925.s006.docx]

Response to Reviewers

| Comment | Response | Revision Location |
| --- | --- | --- |
| Academic Editor: |  |  |
| Please provide more details about the manual classification names that Genderize.io reported with <70% certainty (n=151) and failed to identify (n=90) - which gender suggested Genderize (based on the highest probability) and which you identified. | In the manuscript, we have supplied additional details about the manual classification of names that Genderize.io reported with <70% certainty and failed to identify. We have also added additional information into our supplementary data files. | Methods: Page 7 |
| Please extend the description of Institutions identification in the "Methods". Particularly, include the information on how many Institutions were not in the Times Higher Education World University Rankings. | We have extended the description of institution identification and added the number of institutions that were not listed in the Times Higher Education Ranking in the results section. | Methods: Page 8  Results: Pages 12-13 |
| One of the reviewers pointed out a very important consideration about management of Institutions information by PubMed before and after 2013, this certainly should be described in the "Methods" and considered in the manuscript. | We greatly appreciate the reviewers attention to detail and their expertise. Upon closer inspection of our methods, we realized that we did not accurately describe that the metadata for the deposited data set that we used was from both PubMed and Web of Science. We returned to the data set to ensure that the metadata across the data set included institutional affiliation for all authors regardless of when they published their knowledge synthesis.  For example, our study includes a 2009 study by Mann, Gordon and McLeod and in our data set we can identify that they are located at Dalhousie University, University of Sydney and University of South Australia. We additionally include a 2016 study by Bates and Ellaway that indicates that the authors are affiliated with the University of British Columbia and University of Calgary.  We have updated the manuscript to reflect this clarification. We have also updated the deposited data on Zenodo to include a new column “All Authors Locations”. This update generated a new DOI: 10.5281/zenodo.5088960 | Methods: Page 8  Zenodo: https://zenodo.org/record/5088960#.YOjNrndKicY |
| Please report the exact PubMed search strategy (in Appendix) by which the dataset has been obtained. | The exact search strategies for PubMed and Web of Science are reported in an appendix. | Supplemental Appendix A: List of Core Journals and Search Strategy |
| Please clarify the phrase "Therefore, for authors other than the first author, we report for each knowledge 172 synthesis all countries represented without regard to an individual’s placement in author 173 order." and explain how this influenced the geographic attribution for the non-first authors. | We have attempted to clarify the indicated sentence. We have also added a second sentence that briefly discusses how our inability to identify each author’s country in relation to their author position limits the claims we can make about non-first authors’ geographic attribution. | Methods: Page 8 |
| The statistically significant difference you reported in the phrase "Knowledge syntheses were authored by slightly more females (n=2047; 50.5%) than males (n=2005; 49.5%) across all author positions (Pearson Χ2=22.02, p<.001)." seems rather surprising taking into account the small absolute differences. In the "Methods" you only reported the tools used, but have not mentioned the statistical test applied. Please report statistical tests in details. Considering the use of p-value, it is allowed in the PLoS, but please report also the measurement of uncertainty where needed. | We agree with Reviewer 1 and have removed the chi-square analysis (and corresponding p-values) from the abstract and the body of the manuscript. Because this is an exploratory, descriptive study, and because we had no *a priori* hypothesis regarding gender and author position, it makes more sense to stick with descriptive statistics, as we’ve done for geographic location and institutional affiliation. | Abstract: Page 1  Methods: Page 9  Results: Page 10  Figure 2: Page 10 (legend) |
| Please explain more in details which limitations could be related to the fact that your "research team is made up of individuals from the US, Canada, and the Netherlands", why it is ironic and privileged, and which "various world perspectives that may be relevant in understanding important author characteristics" are reported in the world literature from other regions. | Thank you for calling this to our attention. We have thought about this statement and realized that it does not contribute to the manuscript as we were unable to articulate how it is ironic and privileged in the context of our study. We have elected to remove these two sentences from the manuscript. | Discussion: Page 19 |
| At the Figure 1 you reported "Average" number of authors, indicating the mean. Apart of the terminology, the parametric description does not fit well with the highly skewed distribution of number of authors. Please use instead the median and percentiles (the exact percentile is upon your choice) to present these findings. | We have updated Figure 1 to use the median and the 90th and 10th percentiles | Figure 1: Page 9 |
| Please provide more information about the Core medical education journals listed in the Supplemental Appendix A, with the major aim to justify their selection for the analysis. | We have expanded our description of the core medical education journals. | Methods: Page 6 |
| Considering that the gender is one of the major topics of your manuscript, please present full first names of the authors included in the analysis and listed in the Suppl B. | We have updated the supplement to include full first author names. | S2_Appendix (formally Supp B) |
| You have performed an excellent and very elegant work. The vast majority of classifications have no doubts. However, there are several cases that looks to be classified in a different way. In the Appendix C you have provided full list of names and classifications (and I congratulate you with the adherence to the open research and full reporting!), and there a person having the name "Ashley" is classified as "No data on gender". There are oriental names that you classified as one-gender, while they used for both male and female name - for example Xiao (google the "Xiao male or female name") or Su (google the same or see <https://genderchecker.com/pages/search-engine>). I understand that identification of the gender could be made with knowing additional details you explained in the "Methods" (Linkedin search, etc) and that you can identify the exact gender for a given author with unisex name. Considering this, could you please introduce another column and indicate in the Appendix C for each author whether the gender was identified by Genderize.io or by manual search. | We have added another column in the appendix that indicates for each author whether the gender was identified by Genderize.io (Column D) or by manual search (Column E). | S3_Appendix (formally Appendix C) |
| Please provide some explanations in the tables' footers to make the tables easier to interpret, for example the table in the Appendix E has a column "Count of KS across all author positions" that requires some brief explanations in the appendix (without a need to look at the main manuscript). | We have reviewed all supplemental files and made the following edits:  For S2_Appendix we updated the column headers;  For S4_Appendix_ we added a footnote that contained the World Bank definition of world regions;  S5_Appendix now has two footnotes to define classifications and the “Count of knowledge syntheses (KS) across all author positions”. | S2_Appendix  S4_Appendix  S5_Appendix |
| The figure 3 is very nice, and represents interesting findings. It would be worth it to consider in the "Discussion" the possible reasons for the steep increase in female:male ratio during the short period from 2009 to 2012, and indicate in the "Results" whether these increase has been detected in all groups of countries, or limited only to high-income countries. | Thank you for this compliment of Figure 3. We have added to the discussion information about the potential reasons for the increases and have provided a reference. While we agree that it is worth considering and have done so, we believe it is beyond the scope of the present study to analyze whether this increase has been detected across countries. | Discussion: Page 15 |
| Additional Requirements from Journal: |  |  |
| Please ensure that your manuscript meets PLOS ONE's style requirements, including those for file naming. The PLOS ONE style templates can be found at  <https://journals.plos.org/plosone/s/file?id=wjVg/PLOSOne_formatting_sample_main_body.pdf> and  <https://journals.plos.org/plosone/s/file?id=ba62/PLOSOne_formatting_sample_title_authors_affiliations.pdf> | We have adjusted all file names and in-text formatting to adhere to journal formatting guidelines. | All supplemental and figure file names and manuscript section headings. |
| Please clarify whether this publication [prior work attached to original submission and mentioned on cover page of original submissions] was peer-reviewed and formally published. If this work was previously peer-reviewed and published, in the cover letter please provide the reason that this work does not constitute dual publication and should be included in the current manuscript. | In this cover letter, we have clarified that the related publication submitted in our initial submission has been formally published in *Perspectives on Medical Education* (10.1007/s40037-020-00626-9). We have also noted that we do not believe this current work constitutes dual publication as both papers have different research aims and that for this current manuscript we expanded the data set by adding related data from genderize.io, the Times Higher Education Ranking, and the World Bank’s World Region Classification. | In this cover letter. |
| We note that Figure 4 in your submission contain map images which may be copyrighted. All PLOS content is published under the Creative Commons Attribution License (CC BY 4.0), which means that the manuscript, images, and Supporting Information files will be freely available online, and any third party is permitted to access, download, copy, distribute, and use these materials in any way, even commercially, with proper attribution. For these reasons, we cannot publish previously copyrighted maps or satellite images created using proprietary data, such as Google software (Google Maps, Street View, and Earth). For more information, see our copyright guidelines: <http://journals.plos.org/plosone/s/licenses-and-copyright>. [NOTE: more details in e-mail] | We used Tableau Desktop 2020.4 to develop these maps. This is a paid software that we have access to, similar to Microsoft Excel, and as such, these maps can be included in publications licensed under a CC BY 4.0 licence. | N/A |
| Please include captions for your Supporting Information files at the end of your manuscript, and update any in-text citations to match accordingly. Please see our Supporting Information guidelines for more information: http://journals.plos.org/plosone/s/supporting-information. | Captions have been added to the end of the manuscript and in-text citations are updated to reflect new file names that adhere to PLOS guidelines. | Page 24 |
| Reviewer One: |  |  |
| Overall, I found the article interesting, well-written, and well-informed. Therefore, to start with, I do recommend its publication. Please consider my following comments as an opportunity to improve the manuscript and introduce more clarity where I believe it needs it. | Thank you for your kind works and constructive feedback. | N/A |
| TITLE: |  |  |
| I find the study design lacking in the title, which had me second-guessing for quite a while what was done. To avoid this, I suggest including the term “meta-research”. My suggestion, then, would be as follows:  “Knowledge syntheses in medical education: A meta-research examining author gender, geographic location, and institutional affiliation”  I don’t think it necessary to reference the prolific output by John Ioannidis on the topic of meta-research, but if the authors wish to follow up on this, there are several good articles published in Plos One by him. | We have edited the title to read: Knowledge syntheses in medical education: Meta-research examining author gender, geographic location, and institutional affiliation | Page 1 (title)  Methods: Page 6 |
| METHODS: |  |  |
| The authors say they have done a case study. While this is not incorrect, I think a better description of the study design is meta-research or research on research. | We have changed the description to meta-research. | Methods: Page 6 |
| Lines 131-132  I think you should state clearly from the start that the 14 core journals you used are all indexed in PubMed/MEDLINE, making the “PubMed metadata” phrase a bit less cryptic for readers less versed in bibliometrics. | We have added that all titles are indexed in PubMed/MEDLINE | Methods: Page 6 |
| Lines 143-145  The sentence “Specifically,…” is not supported. Please delete. Additionally, it is repetitive. | This word has been removed. | Methods: Page 6 |
| Line 147  “Data enrichment” sounds strange to my ears. Why not “variables”? | We agree with the reviewer and have removed this sub-heading as we feel that the prior sub-heading data collection also applies to this section of the manuscript. | Methods: Page 7 |
| Lines 158-161  Please consolidate the information provided for the 151 uncertain genders with the 90 not identified, as the same approach was used. | We have consolidated these two sentences. | Methods: Page 7 |
| Lines 171-173  I did not understand this sentence. | We have modified this sentence for clarity. | Methods: Page 8 |
| Lines 186-187  When you use the expression “multiple affiliations,” please specify that you refer to multiple institutional affiliations (as in universities). | We have made this clarification. | Methods: Pages 8-9 |
| Lines 193  An analysis plan is lacking. In my view, this is a strictly descriptive study. You did not do a pre hoc sample size calculation. You used the whole population of knowledge synthesis articles for a set of journals, which is fine. However, you cannot make any generalizations from your data. You should only use descriptive statistics. You have no hypothesis to test. | Thank you. We agree and have removed the chi-square analysis (and corresponding p-values) from the abstract and the body of the manuscript. Because this is an exploratory, descriptive study, and because we had no *a priori* hypothesis regarding gender and author position, it makes more sense to stick with descriptive statistics, as we’ve done for geographic location and institutional affiliation. | Abstract: Page 1  Methods: Page 9  Results: Page 10  Figure 2: Page 10 (legend) |
| RESULTS: |  |  |
| Please remove any p-values from your results. | Thank you, we agree. | See Abstract and Results, para 1. |
| A comment on Geography. You say in methods that you will use the World Bank world region classification system based on the country’s gross national income. I think this indicator is profoundly misleading. Lacking a better one to offer, I understand that you would use it. But maybe consider including in the discussion that many HICs are profoundly different from the countries most represented in your findings (not unsurprisingly, US, Canada, and the UK). Many HICs are more like your LMIC. I consider Global South and Global North a more meaningful description of these differences; although inaccurate, it does place a key emphasis on power relations and asymmetries. | Thank you for your thoughtful response, while we can understand your concerns about the use of the World Bank designations we have decided to retain it. As the reviewer points out, there is no obvious better substitution, and we feel that this indicator will be familiar to many readers.  We have considered the reviewer’s suggestion to include a discussion of how high income countries can vary widely. However, we feel this discussion is beyond the scope of this manuscript. | N/A |
| Line 284  Please specify the country of the National Health Service mentioned. | We have added that this National Health Service is in the United Kingdom | Results: Page 13 |
| DISCUSSION: |  |  |
| Please include a paragraph summing up the main findings before beginning the discussion. | We have added a brief paragraph summing up the main findings | Discussion: Page 14 |
| Line 339  Should say “author” not “authors”. | We have made this correction. | Discussion: Page 16 |
| Line 356  Reference [20] is to the Cochrane handbook and seems out of place here. I would suggest deleting the whole sentence on waste and bias as it distracts readers from the main thrust of the discussion: the concentration of knowledge synthesis in medical education in some (few) countries (that have in common not only English but also wealth). | We have removed this sentence. | Discussion: Page 16 |
| Lines 394-396  Author characteristics not only impact conduct and conclusions but, first, and most importantly, the research questions. | We have added that author characteristics can also impact the formulation of research questions. | Discussion: page 18 |
| Reviewer Two: |  |  |
| Your article is fluent and well-organized. Your figures are clear and helpful. | Thank you for these positive comments. | N/A |
| However, in their current form, your manuscript and the supporting appendices do not supply adequate information about your dataset. I am hoping that you checked all the affiliation data with the article webpages and just failed to make this clear in the manuscript. Based solely on the information you have supplied, I would believe that the author affiliation information in your dataset, for most of the journals studied, was drawn entirely from PubMed. You do not comment on the fact that MEDLINE indexing of authors' affiliations changed in 2013 or describe how you dealt with this change (see "Author, Corporate Author, and Collaborator Affiliation Display Changes." NLM Tech Bull. 2013 Nov-Dec;(395):e9.) . As you probably know, records indexed prior to 2013 were edited by the indexer to include the first author's affiliation only. For example, -- Cook DA, Bordage G, Schmidt HG. "Description, justification and clarification: a framework for classifying the purposes of research in medical education." Med Educ. 2008 Feb;42(2):128-33. doi: 10.1111/j.1365-2923.2007.02974.x. Epub 2008 Jan 8. PMID: 18194162. -- is indexed with Cook's Mayo Clinic affiliation only. Bordage's University of Illinois at Chicago affiliation and Schmidt's Erasmus University affiliation are not reflected in the PubMed record. If your affiliation data for most journals studied were derived solely from PubMed records, your data collection for 1999-2012 articles is seriously flawed. Even PubMed affiliation data for late 2013 - 2019 publications are not entirely trustworthy. The affiliation data are entered by the publisher and are not edited by PubMed's indexers. If the publisher enters the first author's affiliation only, that is the only affiliation reflected in PubMed. For your study, use of records from a literature database that focuses on affiliation indexing (like Scopus) would have made more sense. For an article about the effect of the indexing change on affiliation studies, see Ibarra, M.E., Ferreira, J.P., Torrents, M. et al. "Changes in PubMed affiliation indexing improved publication identification by country." Scientometrics 115, 1365–1370 (2018). <https://doi.org/10.1007/s11192-018-2714-x> | We greatly appreciate the reviewers attention to detail and their expertise. Upon closer inspection of our methods, we realized that we did not accurately describe that the metadata for the deposited data set that we used was from both PubMed and Web of Science. We returned to the data set to ensure that the metadata across the data set included institutional affiliation for all authors regardless of when they published their knowledge synthesis.  For example, our study includes a 2009 study by Mann, Gordon and McLeod and in our data set we can identify that they are each located at different universities: Dalhousie University, University of Sydney and University of South Australia. We additionally include a 2016 study by Bates and Ellaway that indicates that the authors are also affiliated with different institutions: University of British Columbia and University of Calgary.  We have updated the manuscript to reflect this clarification. We have also updated the deposited data on Zenodo to include a new column “All Authors Locations” | Methods: Page 8  Zenodo: https://zenodo.org/record/5088960#.YOjNrndKicY |
| You frequently refer to the Cochrane Handbook. I feel that the Cochrane Handbook would be a more appropriate source of guidance if you were discussing reviews of therapeutic interventions and diagnostic techniques, the types of reviews published in the Cochrane Database of Systematic Reviews. Representation of research from around the world is relevant to reviews of therapeutic interventions and diagnostic techniques. As you note, educational techniques may be more culture-specific. You mentioned the fact that appropriate feedback methods vary depending on the culture. Is this, perhaps, a point in favor of production of culture-specific syntheses rather than a point in favor of including all voices in a given article? Authors and readers want to focus on information that is relevant in their setting. If author/reader time and article length were unlimited, many educators would want to know everything about all cultures and educational systems, but sadly both time and article length are typically limited.  The Cochrane Handbook would be a more appropriate guide if you were discussing articles in journals with essentially no article length limits. As articles in the Cochrane Database of Systematic Reviews are often very lengthy, there is room in these reviews to explore the delivery of care in nations with different economic resource levels. I do agree that information and voices from around the world are relevant to many educational research topics. I also think that many reviews must be focused on educational systems and/or cultures similar to the authors' to accomplish the authors' objectives within many journals' word-number limits ( even when awareness of other cultures and systems would be helpful to educators who deal with students who have come from other cultures). | As we have modified our discussion, we now reference the Cochrane Handbook only twice in the manuscript.  While we can appreciate the reviewer’s thoughts on the inclusion of the Cochrane Handbook, we respectfully disagree that the Cochrane Handbook is more appropriately geared towards clinical topics and have decided to retain this reference. Additionally, in medical education, this resource is frequently used by researchers to guide their knowledge syntheses. Lastly, in reference to page limits, it is true that Cochrane Reviews are quite lengthy, however, medical education knowledge syntheses are published in a variety of journals with varied word limits. | Discussion: Page 18 |
| You mention the growth in number of authors per article and provide some possible reasons for this growth. You haven't mentioned the rapid growth in the publication of systematic reviews and meta-analyses as one reason for this change. Guidelines for production of systematic reviews/meta-analyses require the participation of a multi-author team. | Thank you for this suggestion, we have added that the rapid growth in publication of systematic review and meta-analyses as well as the availability of guidelines that require multi-author teams. | Discussion: Page 14 |
| Your citation 20 on line 97 refers to the entire Cochrane Handbook. It would be better to cite specific chapters, as you have in other in-text citations. Your reader should not have to comb the entire Handbook to find the source of your statement. | To facilitate the reader experience, we have added the specific chapter in the bibliography. | Bibliography: Page 20 |
